# Introduction to Fundamental AI and Ethical Considerations: A Didactic and Interactive Case-Based Session for Preclerkship Medical Students

**DOI:** 10.15766/mep_2374-8265.11645

**Published:** 2026-10-23

**Authors:** John M. Cunningham, Sadie Trammell Velasquez, Luyang Jin, Jason Morrow, Sylvia Botros-Brey, Ronald Rodriguez

**Affiliations:** 1 Assistant Dean for Preclinical Curriculum, Department of Medicine**,** University of Texas Health San Antonio, Long School of Medicine; 2 Director of the Synthesis Curriculum, Department of Medicine, University of Texas Health at San Antonio, Long School of Medicine; 3 Clinical Skills Module Co-Director, Department of Medicine, University of Texas Health San Antonio, Long School of Medicine; 4 Medicine, Behavior, and Society Module Co-Director, Department of Medicine, Division of Palliative Medicine, University of Texas Health San Antonio, Long School of Medicine; 5 Medicine, Behavior, and Society Module Co-Director, Executive Director of the Academy of Educational Scholars, Department of Urology, Department of Medical Education, University of Texas Health San Antonio, Long School of Medicine; 6 Director of the MD/MS in AI Dual Degree Program, Department of Urology, Department of Medical Education, University of Texas Health San Antonio, Long School of Medicine

**Keywords:** Artificial Intelligence, Ethical AI Use, Prompt, Engineering, Case-Based Learning, Ethics, Bioethics, Program Evaluation

## Abstract

**Introduction:**

Widespread integration of artificial intelligence (AI) into health care systems and evolving policies are altering how we must teach medical students. Fundamental AI knowledge and the ethical and responsible use of AI are currently suggested competency domains for health care professionals. Moreover, ethical AI disclosure is incorporated into developing institutional and state policies. Addressing this curricular gap, we created a session to teach preclinical medical students essential AI terminology, prompting strategies, and ethical considerations for AI use in patient care.

**Methods:**

We implemented a 1-hour didactic lecture followed by a 2-hour case-based learning session in the 2025-2026 Medicine, Behavior, and Society module for 236 first-year medical students. We used a postsession, retrospective survey to assess students’ perceived knowledge of the learning objectives. We used the Wilcoxon signed rank test (*\ualpha* = .05) to analyze survey response data. We developed a 5-question multiple-choice quiz (MCQ) to assess students’ postsession knowledge and performed a psychometric item analysis.

**Results:**

Ninety-seven (41.1%) participating students completed the postsession survey and quiz. Postsession perceived knowledge improved across all objectives, with a large (*r* > 0.50) effect size on a 5-point Likert scale. The mean (SD) postsession MCQ score was 3.77 (1.02), with discrimination indices ranging from 0.154 to 0.692.

**Discussion:**

This combined didactic and case-based session delivers timely instruction on AI fundamentals and ethics for preclinical learners. The session significantly increased perceived knowledge, equipping students for future ethical and responsible AI use during clerkships.

## Educational Objectives

By the end of this activity, learners will be able to:

1. Define artificial intelligence (AI), natural language processing, and machine learning.
2. Explain best practices for generating prompt instructions to attain the desired output.
3. Summarize the factors that influence the quality of data and explain how they impact the outputs of AI-based applications, including how training datasets may result in biased outputs.
4. Examine key ethical issues for clinicians, patients, and populations raised by integrating AI applications into the health care system.
5. Recognize that clinicians are responsible for all patient care decisions and exercise judgment in applying AI-generated recommendations.

## Introduction

The introduction and widespread adoption of artificial intelligence (AI) have transformed *what* and *how* we must teach learners to ensure ethical and responsible clinical AI use.^1,2^ Trainees require an understanding of the ethical issues related to clinical AI applications, including bias, transparency, patient autonomy and consent, and data privacy, in preparation for future clinical practice. Real-world cases demonstrate the potential for patient harm when these ethical concerns are not fully understood or misapplied. Examples include biased AI outputs occurring when specific populations are underrepresented in training datasets, such as AI tools for skin cancer detection underperforming on patients with darker skin tones or bias against Black patients when algorithms used health care expenditure as a proxy for health-associated risk.^3,4^ Issues related to the transparency of datasets have led to discrepancies between reported and real-world performance of AI clinician support tools.^5^ Additionally, several states require disclosure of AI technology used in patient care.^6,7^ Clinician AI use without appropriate awareness or knowledge of these known ethical concerns risks adverse or inequitable patient outcomes and potential clinician liability.

The result is a need to train medical students across a range of ethical issues pertaining to AI use, including informed consent, data privacy, transparency (of how applications are used and trained), and patient safety. Initial work identifying competency domains currently guides medical educators’ development of dedicated AI instruction.^8^ Social and ethical implications of AI use and basic knowledge of AI are among the 6 suggested competency domains;^8^ these domains can serve as foundational knowledge for downstream responsible clinical AI use. The preclerkship curriculum offers an opportunity to teach ethical AI practices before students begin using these tools during clerkships, and it is an ideal setting to teach foundational AI concepts.^9^

Since 2023, 4 scoping reviews have examined AI in medical education.^10-13^ Simoni et al^12^ discuss the need for staged integration of initial foundational concepts, such as prompting, followed by later application in the clinical setting. Weidener and Fischer's review^14^ of AI ethics showed broad agreement that ethical training is an essential aspect of digital literacy. The authors suggested grounding AI ethical concepts, such as transparency and data privacy, in the fundamental principles of medical ethics by Beauchamp and Childress,^15^ and recommending small-group, case-based learning formats. However, many of the articles cited were theoretical, and few empirical studies evaluated curriculum content, teaching modalities, program evaluation, or student outcome data.^13^ To date, most evaluations of AI curricula emphasize foundational machine learning and AI training for medical imaging. These published curricula typically focus on elective, rather than required, courses for students.^9-12,16^

As we consider previous work on potential AI competencies within our aim to develop preclerkship (phase 1) AI curricula, we also consider where students are in their training and what specific knowledge prepares them for the next step in their medical education.^17,18^ A recent ethics curriculum by Fernandes et al^19^ provides a valuable interactive session teaching AI ethics through case discussion. We view our work as complementary, with similar coverage of AI ethics. Fernandes et al^19^ detail in greater depth ethical issues surrounding AI systems' potential impact on systems of care and health care access decisions without human oversight. We developed a session in which students directly interact with AI tools using previously learned prompting techniques, fundamental AI concepts, and subsequent AI outputs to work through a case vignette introducing ethical dilemmas. We aimed to introduce preclerkship students to these topics prior to clinical clerkships so they would have the knowledge and skills to prompt a large language model (LLM) to ask a clinical question using recommended frameworks and prerequisite knowledge of ethical considerations required for responsible clinical AI use. We used a didactic approach to teach fundamental terminology, followed by a hands-on case-based learning session to engage students in active, applied learning.^17,20^ While designed for medical students, we believe this session may also benefit faculty and other learners unfamiliar with the implications of newly integrated state laws for clinicians using AI tools in clinical practice.

## Methods

### Session Development

This session was developed by members of the Academy of Educational Scholars, a community of practice for health professions education scholars, in conjunction with the director of the University of Texas Health at San Antonio's dual degree MD/MSAI program, which provides an additional 1-year master's degree in artificial intelligence integrated into a 5-year MD program. Our institutions’ undergraduate medical education (UME) leadership reviewed the content. Faculty content experts developed learning objectives (LOs) to align with existing published competency domains.^8^ Our internal needs assessment highlighted a need to contextualize AI knowledge with hands-on use cases, increased focus on prompt generation, and avoiding the “overly technical,” as such teaching was available within the MSAI program or elective coursework. This aligned with existing literature, suggesting a greater need for students to understand best practices for developing prompts to effectively engage with AI tools.^20,21^

This session was piloted during the 2024-2025 academic year, and the session and evaluation materials were revised based on this pilot. We used a breast cancer screening case vignette to integrate our case-based discussion of ethical issues. The case was developed in collaboration with an oncologist with expertise in breast cancer. We acknowledge that preclerkship students at this phase would lack nuanced case-specific knowledge. The rationale for breast cancer as our AI use case is that there are established guidelines with recommendations summarized in concise text boxes that we could provide students (aiming to reduce student time/effort learning breast cancer screening and allow more time to focus on the AI learning objectives); additionally, breast cancer screening is clinically relevant across multiple specialties and offers diverse potential for small-group discussion (biased AI outputs based on training datasets, contextualizing AI outputs for screening recommendations, transparency and consent for AI use, and human oversight). We present the materials and results for the 2025-2026 academic year. Our session design and outcomes are reported using the FACETS (Form, AI use case, Context, Education Form, Technology, and SAMR) framework. The SAMR framework defines 4 levels of classroom digital technology integration (Substitution, Augmentation, Modification, and Redefinition).^10^ The University of Texas Health at San Antonio deemed this scholarly activity to be exempt from further review (STUDY # 00001426).

### Session Content Outline

This curriculum is intended to be delivered over 3 hours, depending on availability to first-year medical students. The session is split into 2 components: first, a didactic session (50-60 minutes) and, second, an interactive, small-group, team-based learning session using an LLM (90-120 minutes). Within the SAMR, large language models significantly *modify* traditional case-based learning exercises by incorporating and integrating AI-generated outputs.^10^

1. The didactic, given to all first-year students at once as a lecture, introduced strategies for prompting using the TRACI (Task, Role, Audience, Content, and Intent) framework, designed to help users template structured AI inputs to optimize AI outputs.^22^ TRACI was selected as a framework used on the AAMC AI resources page for medical educators. The didactic also covered fundamental terminology explaining LLM function and concepts such as “zero-shot” and “few-shot” prompting and hallucinations (confabulations) (Appendix A). This session also introduced ethical considerations integrating AI into health care systems, including how ethical AI use intersects with the 4 primary components of medical ethics (autonomy, beneficence, nonmaleficence, and justice).
2. The interactive, case-based session presented a clinical vignette on breast cancer screening as an AI use case for hands-on application (Appendices B-D). Students could select a large language model or an AI tool of their choosing to complete exercises during the case. Exercises reinforced key terminology regarding ethical AI use, including transparency, bias, informed consent, and data privacy. The final exercise included built-in debriefing questions to facilitate discussion, including student reflections on skill preservation. Students are in groups of 12 to 16 with 1 faculty facilitator assigned to each group.
3. Postsession, students completed 1 voluntary 5-question multiple-choice quiz and a survey to assess perceived learner knowledge and attitudes pre- and postsession (Appendix E).

### Curricular Context and Session Implementation

Our curriculum is organized into block organ system–based modules. Once weekly throughout each module, students participate in the Synthesis Case Curriculum, a facilitated, case-based small-group session pertaining to the module's weekly content theme. The Synthesis Case Curriculum has 3 goals: provide clinical application of weekly module content, enhance vertical and horizontal integration of curricular material, and foster early development of clinical reasoning and clinical problem-solving skills. The Medicine, Behavior, and Society module covers physician ethics, professionalism, and systems of care. We delivered the AI didactic and small-group session once, as a single scheduled Synthesis session during the Medicine, Behavior, and Society module. While allowing students and faculty to use an LLM of their choosing may increase session variability for individual learners, we embraced this as part of our instruction to meet the learning objectives. For example, students seeing subtly different AI responses from peers based on prompting strategy reinforces the importance of structured prompts (LO 2) and physician responsibility (LO 5) over final decision-making.

Faculty development was essential to standardize small-group, case-based learning. The case facilitator guide (Appendix B) included key definitions and links to current institutional and state AI policies relevant to the discussion. Synthesis faculty facilitator is a paid position in our curriculum, and facilitators can come from any medical specialty and facilitate sessions for their group for the entire year; therefore, the Synthesis course includes an optional 30- to 60-minute facilitator preparation session prior to each session in which a content expert describes the learning objectives, the case, and their approach to small-group discussion questions. We included our AI ethics facilitator prep session immediately before the session didactic, and faculty facilitators were encouraged to attend the didactic session.

### Evaluation Strategy

We used a single-group (no control), retrospective pre-post test design for program evaluation. We developed a 5-question multiple-choice quiz (MCQ) to test learners' knowledge, followed by a survey assessing learners' attitudes toward their knowledge and confidence in applying best practices in prompt generation and ethical principles of AI use in patient care (Appendix E). The survey was created in Qualtrics, an online survey software platform. These 5 MCQ items were internally piloted by faculty within the Division of Internal Medicine and revised based on their feedback and changes to state law and institutional governance policy. We reported item analyses for similar questions in the graduate medical education (GME) setting and made additional revisions as appropriate to trainee level and learning objectives.^23^ The quiz and survey were voluntary and administered via a QR code embedded in the presentation, on the course webpage, and by email. The 5 survey items assessed each learning objective using a 5-point Likert scale (for 3 questions: 1 = not knowledgeable at all, 2 = slightly knowledgeable, 3 = moderately knowledgeable, 4 = very knowledgeable, 5 = extremely knowledgeable). For 2 survey items, we used a 5-point scale (1 = strongly disagree, 5 = strongly agree). We included 1 item that assessed overall session value and an open-ended response item for free-text session feedback. Survey responses were voluntary and anonymous, and MCQ scores did not contribute to learner grading or assessment. We used the Wilcoxon signed-rank test (*\ualpha* = 0.05) to compare presession and postsession survey data. We performed an item analysis on the 5 postsession MCQ quiz items. Data were analyzed using R within the RStudio package.

## Results

There were 236 participating students, with a response rate of 41.1% (n = 97). We did not collect demographic data for this anonymous survey. Students reported significantly higher perceived knowledge postsession compared with their retrospective presession perceived knowledge across all learning objectives (Table 1). A large effect size was observed for all 5 learning objectives (*r* = 0.71-0.81).

**Table 1.**
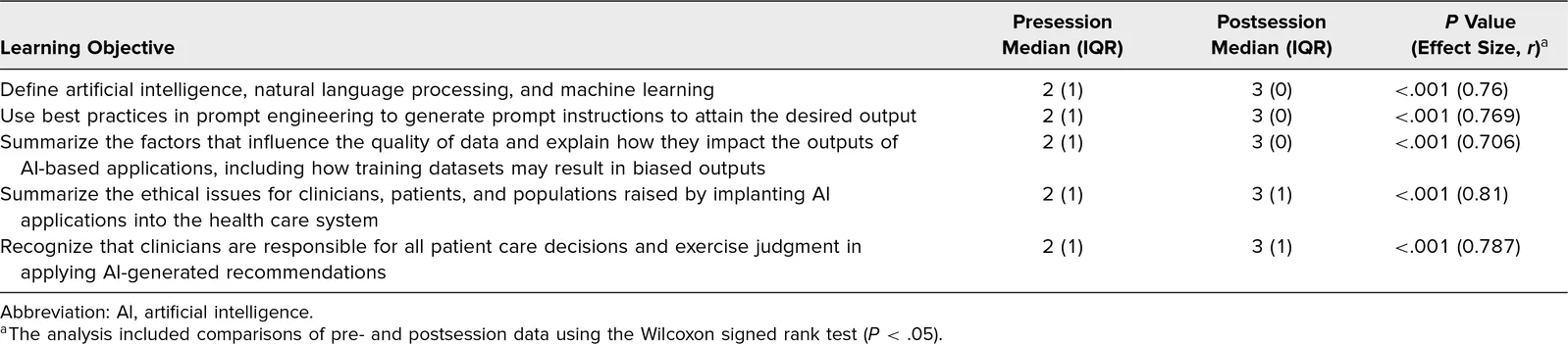
Comparisons of Pre- and Postsession Survey Items of Perceived AI Knowledge (N = 97)

We piloted a 5-question multiple-choice quiz postsession. The mean (SD) score was 3.77 (1.02). Item analyses are presented in Table 2, and distractor analyses are presented in Appendix E. All 5 items had a positive discrimination index (4 of 5 > 0.2), indicating that higher-performing students performed better on these questions. Two questions had discrimination indices approaching 0.7, suggesting possible redundancy in their ability to test the underlying construct. The most challenging question for lower-performing students concerned factors that affect transparency and considerations for integrating AI outputs (LOs 3 and 4) into patient care decisions.

**Table 2.**
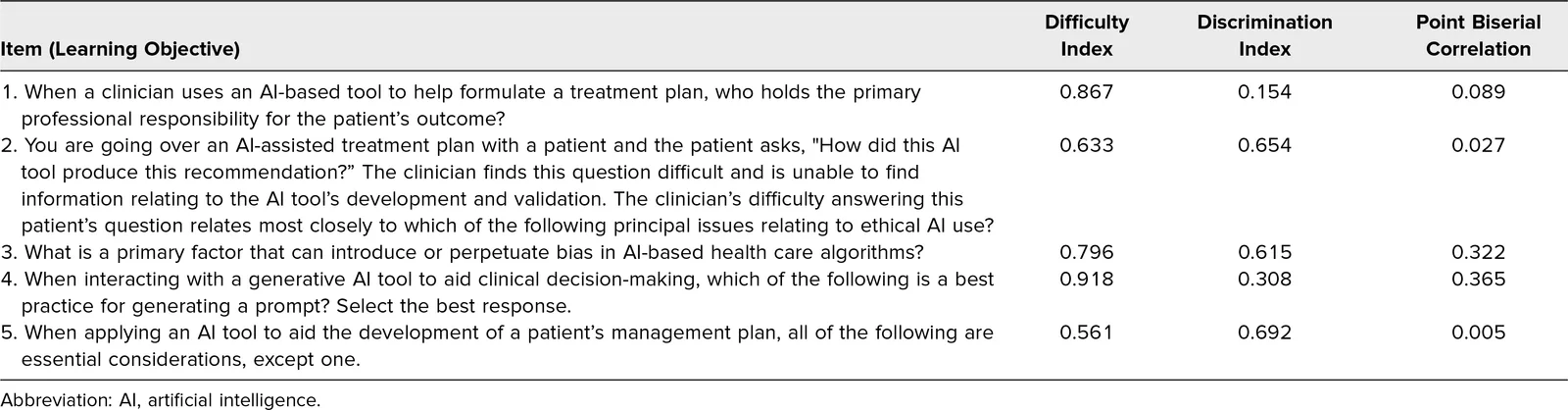
Item Analysis for 5-Question Multiple-Choice Postsession Quiz

Themes of open-ended qualitative feedback included appreciation for learning prompting techniques, enjoyment of integrating AI technology with clear future career impacts, and the interactive nature of the session and discussion with facilitators and peers. While many students are using AI, 1 student commented, “I really liked the prompt engineering session because I really didn't know how to efficiently ask the right questions to get a good response,” demonstrating that not all students had prior exposure to structured prompting frameworks. Even if students were familiar with AI from prior personal or educational use, integrating ethical AI use to promote patient safety was appreciated, with 1 student stating, “I enjoyed how in-depth the sessions are in terms of how to apply and safely use AI in medicine.” Several students acknowledged AI's future impact and enjoyed discussing it with facilitators and peers: “I appreciated the current conversation in which AI will impact our careers as physicians.”  Finally, students appreciated the interactive format of the session, with 1 student stating their appreciation of “the interactivity of discussion with my classmates to bounce ideas off with.” The nature of this student's comment also underscores the value of near-peer learning in an interactive format.

## Discussion

Integrating AI ethical standards and fundamental knowledge required to incorporate AI outputs into the clinical encounter will be essential to future practice.^3,8,21^ Additionally, our current state law mandates disclosure of the use of AI technology for diagnosis or treatment.^7^ Other states and institutions either have or are considering implementing similar policies.^6^ This creates a vital demand to educate learners on ethical considerations for responsible AI use. This session introduced fundamental AI concepts and terminology, serving as a bridge to an interactive case-based learning session in which preclerkship students could practice using an LLM to answer a clinical question and explore ethical issues related to disclosure and shared decision-making. We showed improved perceived knowledge across all 5 learning objectives related to the fundamentals of AI use, prompt generation, and ethical considerations in patient care settings.

This session meets a vital need by providing introductory instruction on AI ethics.^13^ We considered our own prior experience and recommendations from others when deciding to make this a combined didactic, followed by a hands-on interactive session.^12,17,18,20,24^ Faculty clinicians have varying perspectives on what constitutes ethical or acceptable AI use, particularly with early learners.^25^ Having an initial didactic that faculty facilitators were invited to and a faculty review session allowed us to define fundamental AI ethical terminology, such as “bias in AI outputs,” “transparency,” and issues surrounding data privacy and Health Insurance Portability and Accountability Act, to standardize the terminology for faculty and, ideally, enrich the potential discussions between students when working through the case activities. As there remain significant “gray areas” regarding ethical AI use and disclosure, we aimed to facilitate a discussion among students to explore these nuances.

The interactive component also enabled near-peer teaching on applying prompting strategies in the case-based application. Prompting alone has been suggested as a fundamental requirement for clinicians using AI applications in the health care setting.^21^ This session's interactive component provided students with exposure to multiple sources for insights to improve their own prompting practices in addition to working through the structured exercises using the TRACI framework.^22^

While our results indicate significant improvements in perceived knowledge aligned with the learning objectives, the postsession responses remain in the midrange of the Likert scale (ie, for several questions, the median response moved from 2 = slightly knowledgeable to 3 = moderately knowledgeable). Our students’ presession responses are consistent with prior work on the AI readiness assessment, indicating that many students feel underprepared to use AI in health care settings and are somewhat anxious about using AI tools.^26^ It could be deemed somewhat aspirational for students to feel “extremely knowledgeable” after 1 session, given the complexity of the topic covered. Our results show improvement but also indicate that more structured teaching is needed. This aligns with our postsession quiz results, which showed a mean score of ∼75% correct. While our item analysis reveals a need to continue revising individual items and distractors, it also highlights the complex nature of the material and the need for further longitudinally integrated AI teaching across the UME curriculum, as well as further validation of assessment items.

The limitations of this session include using a single-arm retrospective pre-post design, which may limit our ability to attribute observed improvements solely to the intervention. Additionally, recall bias may affect students’ perception of presession knowledge and inflate observed effect sizes. Perceived knowledge gains may not accurately reflect student knowledge. While we conducted a postsession knowledge assessment, we did not include pre- and posttesting or delayed recall testing to test retention.^27^ Additionally, we are in a pilot phase of developing MCQs to assess AI knowledge within a rapidly changing landscape, and the questions may require modification as institutional and governmental AI policies evolve. These sessions can be limited by faculty expertise to facilitate the case-based session, and students may be exposed to slight variations in content presentation, which may influence postsession MCQ responses. It is also possible that some facilitators may convert certain interactive components into a more didactic format in some groups, as some students’ open-ended comments requested even more small-group discussion. Future iterations of this work should assess trainee knowledge retention based on evolving AI ethical frameworks and assess longitudinal student skills as observable competency assessments are defined.^28^

Multiple students commented on this session's application to “future careers,” which seemed a notable area of interest for preclerkship students. We are working with the AI student interest group to include “career fairs,” where AI experts across a variety of biomedical fields discuss their careers working with or developing AI applications. The aim is to reduce uncertainty about career selection and to provide opportunities for future student-faculty mentorship.^29^ We also aim to expand curricula longitudinally by integrating sessions focused on the impacts of AI on clinical reasoning. Such curricular design will be informed by the development of AI-related competencies across the UME-GME spectrum.

## Appendices


AI Terminology and Ethical Use.pptxFacilitator Guide.docxStudent Guide.docxFacilitator Small Group Presentation.pptxPostsession Survey.docx
*All appendices are peer reviewed as integral parts of the Original Publication.*
