# Supplementary material for: Introduction to Fundamental AI and Ethical Considerations: A Didactic and Interactive Case-Based Session for Preclerkship Medical Students
Source: MedEdPORTAL. 2026 Oct 23;22:11645. doi: 10.15766/mep_2374-8265.11645 (PMC13691575; doi:10.15766/mep_2374-8265.11645)
Supplement: Supplementary file 1 [file mep_2374-8265.11645-s001.zip › B. Facilitator Guide.docx]

**APPENDIX B: FACILITATOR GUIDE**

**Clinical Topic: Fundamental Artificial Intelligence and Ethical Use of Artificial Intelligence Healthcare Applications**

**Facilitator introduction:**

This session is designed for preclinical medical students. Part 1 of this session is a review of material from the didactic session on the fundamentals of AI, introduction to large language models, and AI ethical considerations. That session covers the basics of prompting and fundamental AI terminology used in this interactive session. This case comes with a PowerPoint presentation containing information about principles and ethics of AI, case vignette information, and guidance on the correct answers for breast cancer screening. Students at this stage may not have prior exposure to breast cancer screening guidelines, so the PowerPoint and available guidelines allow students to follow along and focus on the AI use and interactive portions of the session rather than medical knowledge. This case follows a patient through their diagnosis of a breast nodule, using breast cancer screening as a lens to discuss the ethical implications of a) AI use in forming care plans and reviewing available evidence, and b) employing machine learning algorithms for breast cancer screening.

**Learning Objectives:** (Appendix D, Facilitator PPT slide 2)

By the end of this session, learners will be able to:

1. Define artificial intelligence, natural language processing, and machine learning
2. Explain best practices generating prompt instructions to attain the desired output.
3. Summarize the factors that influence the quality of data and explain how they impact the outputs of AI-based applications including how training data sets may result in biased outputs
4. Examine key ethical issues for clinicians, patients, and populations raised by integrating AI applications into the healthcare system
5. Recognize that clinicians are responsible for all patient care decisions and exercise judgment in applying AI-generated recommendations

**Timeline: Flexible depending on students’ group discussion.**

30 minutes:	Part 1 “Exploring key concepts” which includes a review of fundamental AI terminology and ethical principles (Activities 1)

1 hour:	Part 2 is “Class case discussion” which includes two activities (Activities 2 and 3) to apply an LLM to a clinical case and discuss ethical issues pertaining to the use of AI algorithms for patient care decision making and informed consent

30 minutes:	Part 3 is “Synthesis case conclusion and debrief” and which contains the case outcome and time to debrief on the issues relating to using AI to achieve ethical shared decision making with a patient (Activity 4).

Student prework: Optional

1. [Principles of artificial intelligence and its application in cardiovascular medicine - PubMed](https://pubmed.ncbi.nlm.nih.gov/37721424/)
2. [The need for health AI ethics in medical school education - PubMed](https://pubmed.ncbi.nlm.nih.gov/33655433/)

**Part 1: Exploring key concepts**

Part 1 content derives from the Fundamentals of Clinical AI use lecture (Appendix A).

Prior to the didactic session, the student prework above presents overviews of fundamental AI terminology and ethical considerations that will guide discussion during the activities below.

Activity 1: Discuss the definitions of key terminology and review of a use case from the prework.

1. Students should spend 5-10 minutes in discussion reviewing the following fundamental AI terminology: 1. Machine Learning (supervised and unsupervised learning), 2. Natural Language Processing 3. Deep Learning 4. Artificial Intelligence. The definitions below are designed as a cheat sheet for facilitator review.
2. Describe primary ethical principles when incorporating generative AI and machine learning algorithms into patient care settings?

#1. Definitions of key terms **(Facilitator PPT Slide 3):**

Artificial Intelligence: Broadly, machines that mimic human intelligence to perform problem-solving functions and learning using automation and predictive algorithms to solve complex tasks.

Machine Learning: In typical algorithms (e.g., a pocket calculator), the algorithm receives an input, provides an output, but does not learn. It gives the same output every time. Machine learning involves the use of more complex AI systems that can receive inputs and learn from data; therefore, the algorithm and the downstream content of the output can change over time. The learning system itself can involve neural networks that mimic the human brain (deep learning).

Natural Language Processing: This is the subfield of Artificial Intelligence that uses machine learning to allow computers to communicate with human language. Common examples include Apple’s Siri and Amazon’s Alexa.

Deep Learning: A subclass of machine learning using artificial neural networks (ANN) to replicate brain architecture (neurons are in layers). The most basic component is called a “node.” In ML, a node receives inputs called weights and biases, which it uses to calculate the output it passes to the next layer. Using the example of a mammogram, the image pixels would be the input (initial layer). Characteristics such as calcifications in the pixels or tissue density contribute to the weights and biases (hidden layer) that lead to the output “positive” vs. “negative” screen (output layer). This is compared to the “ground truth” (or actual label of positive vs. negative screen), and the algorithm incorporates whether it correctly labeled it, vs. the error rate. The algorithm can learn as repeated training images are included in the data set. Supervised learning is when clearly labeled mammograms are provided. Unsupervised learning is when the ML algorithm analyzes and detects patterns in the data on its own, using unlabeled data.

#2 Principles of AI Ethics **(Facilitator PPT slide 4-5):**

- **Informed consent** – If a physician uses health AI to develop a treatment plan, whether for diagnostic research or clinical reasoning, does the patient have the need to know, or right to know? If so, is notification sufficient, or should the physician first seek permission? While disclosure and consent may seem unnecessary in most cases—for example, when conducting a general literature search related to breast cancer but not in relation to a specific patient’s medical record—consider the circumstances when they might be necessary. Furthermore, what exactly should be disclosed when disclosure is required? The tool, alternatives, and the possibility of error or hallucinations? Does notice or consent continue to apply to other related therapeutics decisions? Would a general notice at an office or hospital suffice? In direct patient care, agreement upon a care plan is always between humans, not LLMs, so the physician and patient together are the final arbiters of appropriate clinical decisions.

Comment: Clinicians have a duty to disclose information that a “reasonable person” would need to know in order to make a decision. Disclosure should be driven by two considerations: (1) how serious is the risk that using the tool could cause harm (nonmaleficence) and (2) to what extent do patients have a meaningful opportunity to participate in choices about their private information (respect for autonomy)? Many states are passing legislation requiring disclosure and students are advised to research these statutes where they train and practice. Also, if clinicians consult general purpose LLMs (E.g., Claude, ChatGPT, Gemini that are not protected by an institutional firewall or BAA), they should avoid using personally identifiable information; even obtaining and documenting patient permission may not be adequate since the contours of how private information is used or shared by LLMs may not be known to the physician.

- **Bias** – What is the potential for AI to mitigate or exacerbate existing healthcare disparities? Existing bias can be propagated if the AI algorithm is trained on biased data sets (example – an AI algorithm that assigned similar risk to black patients despite black patients in the data set being sicker. This occurred because the data set used healthcare costs as a proxy for the level of health needs (Obermeyer et al, 2019). Similarly, a Dermatology algorithm for identifying skin pathologies only had 4 -18% representation of darker skin tones in the training data sets, leading to misclassification of dark-skinned individuals upon deployment. Underrepresentation of specific populations in training datasets can result in algorithmic bias, reducing the accuracy of AI predictions for the underrepresented patients. **These studies highlight the importance of internally validating data sets and outcomes with representative samples of the local population** before applying AI algorithms.
- **Safety** – Many healthcare AI programs are not FDA-regulated, and safety outcomes are unknown. The FDA is taking steps to apply safeguards to different types of AI algorithms. One issue to consider is how frequently healthcare systems audit their AI algorithms, especially if they use adaptive machine learning algorithms, as their safety profile may evolve over time after initial approval. Consider an initial EPIC sepsis alert algorithm, which was shown to perform under the reported AOC when implemented at institutions in real-world settings. As of now, many of these tools are given the designation “clinical support tool.” Which means they cannot be used to make patient care decisions independently.

Comment: This relates to the principle of non-maleficence—physicians are obligated to FIRST do no harm. AI safety and oversight must be baked in, not added on as an afterthought. We get excited about the potential to improve patient care, but we should strive to first ask, “Do I need this tool?” and reasonably attempt to anticipate all the risks, then create systems to respond to those risks quickly, honestly, and effectively. Like in a car, the brakes may be more important than the gas.

- **Transparency** – Transparency using AI-based technologies often refers to issues pertaining to the “black box” phenomenon. Essentially, many of the layers of deep learning and neural networks are not easily obtainable/describable/or readily available to bedside clinicians. If an AI-based model predicts mortality for a patient based on an algorithm that cannot be explained, and this differs from the clinician’s opinion, how should this be handled/disclosed? In a European meta-analysis of radiology studies, only 11.5% of deep learning models were made available to readers for validation; 22.1% had training coding available. How are you as a clinician supposed to explain training data sets and how these models draw their conclusions?

Comment: This is tricky. Consider: Not all physicians understand the statistics underpinning evidence-based medicine. What if a high-profile and widely accepted study is at odds with an individual clinician’s opinion? Well, they are probably obligated to understand why there is a discrepancy. They should scrutinize the study and either change their opinion or clarify why the discrepancy is not significant or relevant. **Clinicians don’t have to fully understand the layers of neural networks, but they have a duty to reconcile AI answers with the existing standard of care, to always use clinical judgment alongside newly developed clinical tools, and to disclose the role of AI as transparently as able to enable a patient to make an informed decision.**

- **Patient Privacy** – There are a multitude of issues here. **Most simply, students should never enter PHI or identifiers into a 3rd party LLM.** However, on a broader level, there are issues regarding the transfer of data to train various algorithms between healthcare settings. What happens if a program used at one institution is sold to a larger company and changes its privacy and data regulations? What happens if a prognostic tool collects additional data not typically provided by patients (e.g., zip code, income)? What if insurance companies obtain access to this data? Comment: No need to dwell on this particular concern for this session, but it’s a topic worth acknowledging. A patient’s personal data can be packaged and sold to parties unrelated to their clinical care.
- **Authenticity and Humanity** – Physicians should not be a passive vessel for AI clinical reasoning and management. Nor should they be merely in the loop when AI is consulted. Physicians should have both oversight, authority, and creative input into any patient assessment and plan. Accountability for how literature searches and AI consultation is used speaks not only to the duties of the physician—as in, where the buck stops—but also to the need for human beings within intimate, caring relationships. Consider how trust and humanism may evolve in the era of clinical AI. Comment: This is not in the pre-reading, but we would like the students to reflect on the risk AI poses to them as healers, potentially in the debrief. If physicians increasingly rely on AI-based tools to offer assessments and recommendations, what exactly is the physician offering? Couldn’t patients just consult ChatGPT on their own? If AI-based interfaces can generate empathic statements—and patients have positive responses—will physicians hand communication over so they can focus on other things (and what would those things be)? Who will remain accountable for effective communication? We’re curious: do the students wonder or worry about this? If so, what should we—students and educators alike—do to ensure our role as healers?

**For reference:** Verghese A, Shah NH, Harrington RA. What this computer needs is a physician: humanism and artificial intelligence. JAMA. 2018;319(1):19-20.

doi:10.1001/jama.2017.19198.

**Part 2: Class case discussion** **(Facilitator PPT slides 6-7)**

We now transition to the case. A recent NEJM piece provided some insight into “low-stakes” vs. “high-stakes” AI use, with examples. Here we are in simulation using a “case vignette,” so therefore, “low-risk.” Yet, the case vignette clearly simulates high-risk AI use. The purpose here is to have students apply the ethical constructs above and reflect on how they consider their own AI use in such a situation (e.g., looking up information during an early clinical experience). The key element of low stakes: doesn’t impact patient care decisions; lots of time to audit the AI output. High-stakes AI use generally affects patient care orders or decisions, and in situations where there may be minimal time to augment or audit the AI output.

Low-Stakes tasks: practicing with AI case-based simulation (no direct care decisions involved),

ambient AI scribe (time and required human revision required).

High-stakes: using a general-purpose LLM for clinical decision support (impacts patient care orders), using/applying an AI-based ECG or CXR reader (minimal required oversight).

**For reference:** [Educational Strategies for Clinical Supervision of Artificial Intelligence Use - PubMed](https://pubmed.ncbi.nlm.nih.gov/40834302/). Abdulnour RE, Gin B, Boscardin CK. Educational Strategies for Clinical Supervision of Artificial Intelligence Use. *N Engl J Med*. 2025;393(8):786-797. doi:10.1056/NEJMra2503232

**Case:**

A 47-year-old woman presents for initial breast cancer screening. She does not endorse any breast pain, redness, swelling, overlying skin changes or nipple discharge. The patient denies weight loss, swelling in the axilla or neck, or other bone pain. She reports a mammogram 2 years prior in which she was noted to have dense breast tissue. She reports being worried that a mammogram could miss a possible cancer diagnosis.

Past Medical History: menarche at age 15.

Currently on no medications. Used estrogen/progesterone OCPs from age 23-33 years of age.

Surgical history- status post uterine fibroid resection. C-section x 1. s/p Tubal ligation.

Social history – Never smoker. Occasional EtOH < 1 drink per week. Married, has one child.

Family History – mother died of colon cancer at age 62. Father died of lung cancer at age 71. No family history of breast cancer.

Objective:

Vitals: 98.5, HR – 83 bmp, RR- 16 bpm, BP – 105/58 mmHg. 98% on room air.

Gen- well appearing, no acute distress

Neck – no cervical or supraclavicular lymphadenopathy

Breast – no palpable nodules. No overlying skin redness.

Lungs – no use of accessory muscles. clear to auscultation bilaterally.

CV- regular rate and rhythm with normal S1/S2 and no m/r/g

Abdominal – soft, non-distended, bowel sounds present, NTTP in all 4 quadrants. No hepatosplenomegaly

Extremities- no edema

Lymph- no axillary or cervical adenopathy palpated

Activity 2: Case discussion questions: **(Facilitator PPT slide 8)**

1. Briefly, have students discuss structured methods for generating a prompt.
2. Use the LLM to understand key components of the history, physical, and diagnostic tests to assess breast cancer risk.
3. Using best practices in prompt generation – ask the LLM to make a recommendation on the initial mode of breast cancer screening for this patient. If students have access, have them try different LLMs (Open-Evidence vs. Gemini or OpenAI, or others).
4. Guide students through a structured approach to appraise AI outputs using Sackett’s 5-step framework for evidence-based practice.
5. Ask the LLM what references it used to make its decision and verify those references. Did anyone obtain hallucinated references?
6. Is the strength of the recommendation from the AI tool consistent with the guidelines? (guidelines provided at the table, Canvas, or PubMed link below)

This exercise focuses on prompting and learning the strengths, limitations, and ethical issues related to applying AI tools for clinical decision-making. Students will not have the prerequisite knowledge of breast cancer screening per se, and the facilitator PPT (slide 10) and student PPT will provide relevant portions of the guidelines on breast cancer screening for reference to help audit the AI outputs. Students should discuss their results with each other. Listen for variation in AI outputs.

#1 Best practices when prompting **(Facilitator PPT Slide 9):** While there are other frameworks, the TRACI framework provides an example of a structured method for generating a prompt. The reason to consider these structured approaches is to reduce the risk of hallucinations/confabulations that can occur with vague prompts that don’t clearly direct the LLM what information to provide.

**TRACI Framework**

**Task:** What do you want to achieve?

**Role:** What role or persona should the AI adopt? e.g., expert statistician

**Audience:** Who is the output intended for? (reading level, patient appropriate language

**Create:** what content? (text, image, code)

**Intent:** what is the purpose? Inform, persuade

Ask students how they incorporated the case data into the prompt (did they copy and paste vs. summarize the case?). This part has important ethical implications as students enter the wards. **Students should not copy and paste information from a chart into an AI tool unless the institution has identified it as HIPAA-compliant.** Key content should be summarized to generate the prompt. However, this activity itself requires some medical knowledge to highlight the most salient points and is an interesting feature of integrating AI outputs into clinical practice. Essentially, the more you know, the better you can craft a specific prompt and audit the AI output.

#2. Correct responses number 1: History: age at menarche/menopause, OCP use, obstetric history, tobacco/EtOH, family history, size and location of the breast nodule (if any), presence of axillary/supraclavicular LN, and the size/shape/morphology of the breast mass on mammogram.

#3 USPTF statement from 2024 recommends initial 2D digital mammography. **(Facilitator PPT slide 10)** While it acknowledges fewer false positives with 3D digital breast tomosynthesis, it has not yet shown additional benefits in breast cancer detection rates. DBT did not show a mortality benefit over 2D mammography. The expert acknowledges that these are the current guidelines, but there is some disagreement with respect to screening patients with dense breast tissue amongst experts and **recommends Digital Breast Tomosynthesis (DBT).** It is interesting to acknowledge this uncertainty and take it into account when using the LLM (Does the LLM give definitive answers or acknowledge the uncertainty?)

**For reference:** [Screening for Breast Cancer: US Preventive Services Task Force Recommendation Statement - PubMed](https://pubmed.ncbi.nlm.nih.gov/38687503/) *US Preventive Services Task Force, Nicholson WK, Silverstein M, et al. Screening for Breast Cancer: US Preventive Services Task Force Recommendation Statement. JAMA. 2024;331(22):1918-1930. doi:10.1001/jama.2024.5534*

**If unfamiliar with LLMs and wanting to try: here is a sample prompt:

You are an expert Oncologist seeing a 47-year-old female patient in clinic for follow-up breast cancer screening. The patient was noted to have increased breast density on prior screening. Use evidence-based medicine, referencing USPTF, Oncology, and Radiology guidelines to develop an initial screening recommendation. Incorporate appropriate diagnostic tools and treatments as clinically indicated. Cite specific guidelines when applicable.

#4. Sackett’s “5A’s” for evidence-based practice are modified here for incorporating AI use **(Facilitator PPT Slide 11)**. This session is not designed for full integration of how to ask a PICO question. However, students may want an approach – The key here, and bolded below – clinicians should apply skeptical appraisal to ensure they achieve a decision that **meets the standard of care.**

| **EBP Step** | **Application to AI Use** | **Example Clinical Question** |
| --- | --- | --- |
| 1. Ask the question | Formulate a clear, answerable question using a structured prompt | In a 47-year-old female with heterogeneously dense breast tissue, and no family history of breast cancer, what is the optimal test for initial breast cancer screening? |
| 2. Acquire information | Use the AI tool to retrieve relevant evidence, guidelines or summaries. | Summarize and contrast USPSTF, Oncology, and Radiology guidelines comparing recommendations? |
| 3. Appraise | Critically evaluate the AI output for accuracy and alignment with the standard of care. Compare with trusted sources. | Does the AI-recommended strategy match UpToDate or guideline-based recommendations? |
| 4. Apply findings to practice | Integrate the AI-outputs with clinical expertise and patient values. | Would this strategy be appropriate for this patient given their greater fear of a false negative than a false positive? |
| 5. Assess outcomes | Reflect on the outcome and the AI tool’s utility. Consider revising prompts or finding alternative sources. | Did the AI output improve decision-making or require significant correction? Patient outcome? |

Table: *author owned*

#5. After clicking on references – were any references hallucinations in the LLM’s output?

#6. This question may yield some interesting answers – LLMs are trained to make the most likely prediction and don’t always convey uncertainty, whereas guidelines are notorious for conveying where there is a lack of evidence. When I did this activity, the LLM provided very definitive recommendations for DBT based on dense breast tissue, which differed from the USPSTF guidelines. When I asked the LLM to explain its reasoning or “How sure are you,” that follow-up prompt led to a more detailed discussion comparing different guidelines.

What are the implications of the certainty of LLM responses when using LLMs to assist with patient care decisions? This portion is for student practice; have them ask the LLM, “How confident are you in your decision? Do any society guidelines have alternate recommendations? What specific features of this case led you to recommend X diagnostic test?

**Case Continued (Facilitator PPT slide 12)**

Imaging – Digital Mammography and bilateral digital 3D tomosynthesis and a limited US was performed. And synthetic 2D digital mammography images were obtained. Computer-aided R2 CAD.

- 1.4 x 0.8 x 1.0 cm nodule in the left breast at 7 o'clock, which persists on focal compression. BI-RADS IV- suspicious finding. Heterogeneously dense breast tissue.
- On focused US – hypoechoic parallel mass with irregular margins. No significant internal vascularity. US of the left axilla shows normal lymph nodes.

Vignette prior to activity 3: **(Facilitator PPT slide 13)**

Based on the results of a competition to yield improved algorithms for identifying cancerous breast lesions on DBT, your hospital has adopted a machine learning algorithm that predicts malignant vs. benign lesions. The algorithm has a sensitivity of 0.96 for detecting of cancerous lesions on biopsy.

**For reference, optional:** [A Competition, Benchmark, Code, and Data for Using Artificial Intelligence to Detect Lesions in Digital Breast Tomosynthesis - PubMed](https://pubmed.ncbi.nlm.nih.gov/36821110/) *Konz N, Buda M, Gu H, et al. A Competition, Benchmark, Code, and Data for Using Artificial Intelligence to Detect Lesions in Digital Breast Tomosynthesis. JAMA Netw Open. 2023;6(2):e230524. Published 2023 Feb 1. doi:10.1001/jamanetworkopen.2023.0524*

The AI algorithm predicts the patient’s lesion as benign, but the attending's clinical experience leads them to recommend a biopsy in accordance with standard guidelines for managing a BI-RADS IV nodule.

Activity 3: Case (cont.) discussion questions: **(Facilitator PPT Slide 14)**

1. Use a generative AI application of your choosing. How should this patient’s DBT and US be followed up? What additional testing could be performed? What ethical concerns must be addressed when incorporating AI into this patient care decision?
2. When such a machine learning algorithm assists in the interpretation of biopsy results within the local healthcare system, what ethical issues arise? (consider bias, transparency, consent). What factors influence the decision to adopt such tools?
3. How do you inform the patient about any potential discordance between AI and human recommendations for the follow-up plan for the biopsy?
4. Can the LLM adjust recommendations based on patient preference? Try additional prompts that incorporate variations of patient preferences.

Facilitator note: Students at this stage in their training won’t have the knowledge to verify the correctness of AI-based outputs for question 1, or to know if the LLM references reliable sources. Below is a summary of faculty answers, which can serve as “what should happen.” However, use this time to let the students explore ethical issues, potentially have them use the LLM to determine how best to communicate discordant recommendations or transparent AI use, or even deliver the news of the mammography results. If you get through this portion too fast, students could enter additional prompts (e.g., apply the SPIKES to generate a method of disclosing the results of the mammogram to the patient in an empathetic manner)

#1. Generally, a BI-RADS IV lesion is followed up with a core needle biopsy. That is what occurred in this case and was the expert’s recommendation. As early learners, the ethical considerations include whether making such a decision falls within their scope of practice, how they can ensure AI recommendations meet the standard of care, and which sources they should consult to verify AI outputs.

#2. The algorithm was developed at another institution, and adoption of AI algorithms requires internal validation using local data sets with representative patient populations and working with IT to ensure data privacy during the validation phase. Essentially, the biggest issue to address here is the potential for bias; if the training data sets at the outside institution had a different demographic representation, the results (positive and negative predictive values) may not apply to our patient population at UH. Bias in AI outputs could adversely impact patient safety.

There will also be issues regarding data privacy as this patient’s data becomes part of the training set at your institution. Finally, the issue of transparency – how well can you describe how this machine learning algorithm achieved a decision of “benign?” Did the initial data set include sufficient numbers of patients like yours, or could the initial training data set be biased? Does the patient have the right to know that a sensitive algorithm predicted a benign result? Would this impact their decision to move forward with a biopsy? Should it?

Students must also take away that their role in making the final decision is that these algorithms offer clinical support but cannot make the final determination – “Human-in-the-loop” decision-making.

#3.  This is a situation in which disclosure is ethically indicated regardless of the new state laws.

Discussions of discordant recommendations with a patient should center on the “standard of care” and why it is or is not recommended for the patient’s particular case. If a patient holds a value or belief and does not want a follow-up test that the AI tool recommends, what should the provider do? The primary considerations for such a situation are: a) the clinician holds the final responsibility, b) AI outputs should be appraised with respect to the standard of care, c) shared decisions between clinician/patient and clinician/patient/AI should respect patient autonomy, and d) don’t overlook – the treatment plan falls within the appropriate scope of practice for the clinician.

#4 – There is no right answer here, but students can discuss their attempts to generate recommendations based on patient characteristics.

**Part 3: Synthesis case conclusion and debrief (Facilitator PPT Slide 15)**

Part three contains the case conclusion and debriefing session for this synthesis session. What aspects of integrating AI into this case seemed useful? What ethical challenges arose from integrating AI outputs into this patient’s care?

The patient underwent a biopsy, which revealed a Fibroadenoma without signs of malignancy. The patient’s loss of her parents due to other cancer types and young children, as well as independent reading on the impact of dense breast tissue, strongly impacted her own perceptions of risk vs. benefits of additional management. The Surgical Oncologist and Breast Radiologist recommend an excisional biopsy due to the discrepancy between her nodule imaging characteristics and biopsy results.  After a discussion with the Breast Radiologist and Surgical Oncologist, she opts to proceed with an excisional biopsy.

Excisional biopsy results:
• Excisional biopsy- Fibroadenomatoid change. Background sclerosing adenosis and microcalcification. Negative for in situ or invasive carcinoma

Patient has a stable post-op course and is recommended to resume routine screening.

Activity 4: Debrief: These questions are suggestions and additional questions can be generated by the facilitator based on the group’s discussion and interest. **(Facilitator PPT Slide 16)**

1. As first-year students, what was the most useful task the LLM performed? Guidance on the important risk factors and data to collect when seeing the patient? Or the potential to help make individual patient care decisions? Or simply time to read more about breast cancer screening and obtaining reference sources to guide learning?
2. What are your biggest concerns as you navigate the role of managing AI predictions or patient care recommendations? (Do you acknowledge this as being “high-stakes” AI use?). How can we as educators address those concerns?
3. As a student, you may consider using AI outputs both to learn and to assist in generating a care plan. These different roles carry different responsibilities and risks with respect to managing the AI output. Reflect on the slightly different roles. Have you found anything effective when using AI to enhance your own learning as opposed to risking “cognitive offloading” or atrophy of your critical thinking skills?
4. Given AI’s current environmental footprint and power requirements, should AI be used for clinical care recommendations when readily available access to guidelines or clinical risk calculators already exist?
5. Auditing AI output to ensure accuracy and “human-in-the-loop” decision-making takes time; is it worth the time? Or does the efficiency loss of this verification process guide you towards using other reference materials?

#1 – This question is only for reflection. If students get through it too quickly, guide them to put in some of the prompts that could explore the above questions if they did not earlier.

#2 – Lots to explore. Consider the legal implications and whether we will have enough time to audit AI outputs in real-time for patient decision-making. Will AI companies truly and transparently release their validation processes to a degree that lets us clearly inform patients of risks and benefits? Will patients continue to trust my clinical judgment during the shared medical decision-making process? Will healthcare systems and companies prioritize developing AI tools that enable clinicians to function at their best (reduce cognitive load) or create a more distracting clinical environment?

#3 – How do you learn with AI vs. the well-documented risks of cognitive offloading and deskilling? There are (as of yet) no proven strategies to mitigate this risk. Best-practice recommendations for early learners include making the process of auditing AI outputs or reflecting on their own AI use part of an ‘active learning’ process rather than passive learning (to avoid automation complacency).

#4 – This issue is less often discussed but vitally important. AI has immense power requirements. During model training, a single NLP model emits 5 times as much CO2 as the average car emits in its lifetime. A single data center consumes the equivalent of heating 50,000 homes yearly.  At this time, we have little ability to offset these power requirements, and some have commented that the environmental impact falls on those already affected by healthcare disparities.

**Reference, optional:** [AI Code of Conduct-Safety, Inclusivity, and Sustainability - PubMed](https://pubmed.ncbi.nlm.nih.gov/39466265/) *Gonzalez A, Crowell T, Lin SY. AI Code of Conduct-Safety, Inclusivity, and Sustainability. JAMA Intern Med. 2025;185(1):12-13. doi:10.1001/jamainternmed.2024.4340*

#5 – This question has significant areas for exploration with students. We have been in the age of big data for decades. It has long been acknowledged that we need to teach medical students what they “need to know” vs. what can be “looked up.”  However, AI, specifically generative AI, has unique challenges. Previously, if I looked up an antibiotic dose or the workup for a breast nodule using a peer-reviewed source, I accessed that information through a medical reference. Generative AI tools, in and of themselves, cannot serve as a reference; they simply cite other sources, which must be verified before incorporating the output into a care decision. Additionally, if a student uses an AI tool as an educational source, he/she may be “led down the wrong path” by a hallucination, but that only affects them. The stakes are much higher when trying to augment a patient care plan with AI (high vs. low-stakes AI use).
