# Supplementary material for: Introduction to Fundamental AI and Ethical Considerations: A Didactic and Interactive Case-Based Session for Preclerkship Medical Students
Source: MedEdPORTAL. 2026 Oct 23;22:11645. doi: 10.15766/mep_2374-8265.11645 (PMC13691575; doi:10.15766/mep_2374-8265.11645)
Supplement: Supplementary file 1 [file mep_2374-8265.11645-s001.zip › C. Student Guide.docx]

**Appendix C: Student GUIDE**

**Clinical Topic: Fundamental Artificial Intelligence and Ethical Use of Artificial Intelligence Healthcare Applications**

**Introduction:**

Part 1 of this session reviews material from the didactic session on the fundamentals of AI, introduction to large language models, and AI ethical considerations. This case follows a patient through their diagnosis of a breast nodule using breast cancer screening as a lens to discuss the ethical implications of a) AI use in forming care plans and reviewing available evidence, and b) employing machine learning algorithms for breast cancer screening. We understand you have not yet had sessions on breast cancer screening, and the USPTF guidelines and expert recommendations are provided for reference as you explore AI outputs throughout the case.

**Learning Objectives:**

By the end of this session, learners will be able to:

1. Define artificial intelligence, natural language processing and machine learning
2. Explain best practices generating prompt instructions to attain the desired output.
3. Summarize the factors that influence the quality of data and explain how they impact the outputs of AI-based applications including how training data sets may result in biased outputs
4. Examine ethical issues for clinicians, patients, and populations raised by integrating AI applications into the healthcare system
5. Recognize that clinicians are responsible for all patient care decisions and exercise judgment in applying AI-generated recommendations

**Timeline: Flexible depending on students’ group discussion.**

30 minutes:	Part 1 “Exploring key concepts” which includes a review of fundamental AI terminology and ethical principles (Activities 1)

1 hour:	Part 2 is “Class case discussion” which includes two activities (Activities 2 and 3) to apply an LLM to a clinical case and discuss ethical issues pertaining to the use of AI algorithms for patient care decision making and informed consent

30 minutes:	Part 3 is “Synthesis case conclusion and debrief” and which contains the case outcome and time to debrief on the issues relating to using AI to achieve ethical shared decision making with a patient (Activity 4).

Student prework: Optional

1. [Principles of artificial intelligence and its application in cardiovascular medicine - PubMed](https://pubmed.ncbi.nlm.nih.gov/37721424/) Wieneke H, Voigt I. Principles of artificial intelligence and its application in cardiovascular medicine. *Clin Cardiol*. 2024;47(1):e24148. doi:10.1002/clc.24148
2. [The need for health AI ethics in medical school education - PubMed](https://pubmed.ncbi.nlm.nih.gov/33655433/) Katznelson G, Gerke S. The need for health AI ethics in medical school education. *Adv Health Sci Educ Theory Pract*. 2021;26(4):1447-1458. doi:10.1007/s10459-021-10040-3

**Part 1: Exploring key concepts**

Part 1 content derives from the Fundamentals of Clinical AI use lecture.

Prior to the didactic session, the student prework above presents overviews of fundamental AI terminology and ethical considerations that will guide discussion during the activities below.

Activity 1: Discuss the definitions of key terminology and review of a use case from the prework.

1. Spend 5-10 minutes in discussion reviewing the following fundamental AI terminology: 1. Machine Learning (supervised and unsupervised learning), 2. Natural Language Processing 3. Deep Learning 4. Artificial Intelligence. The definitions below are designed as a cheat sheet for facilitator review.
2. Describe primary ethical principles when incorporating generative AI and machine learning algorithms into patient care settings?

**Part 2: Class case discussion**

We now transition to the case. A recent NEJM piece provided some insight into “low-stakes” vs. “high-stakes” AI use, with examples. Here we are in simulation using a “case vignette,” so therefore, “low-risk.” Yet, the case vignette clearly simulates high-risk AI use. The purpose here is to have students apply the ethical constructs above and reflect on how they consider their own AI use in such a situation (e.g., looking up information during an early clinical experience). The key element of low stakes: doesn’t impact patient care decisions; lots of time to audit the AI output. High-stakes AI use generally affects patient care orders or decisions, and in situations where there may be minimal time to augment or audit the AI output.

**Case:**

A 47-year-old woman presents for initial breast cancer screening. She does not endorse any breast pain, redness, swelling, overlying skin changes or nipple discharge. The patient denies weight loss, swelling in the axilla or neck, or other bone pain. She reports a mammogram 2 years prior in which she was noted to have dense breast tissue. She reports being worried that a mammogram could miss a possible cancer diagnosis.

Past Medical History: menarche at age 15.

Currently on no medications. Used estrogen/progesterone OCPs from age 23-33 years of age.

Surgical history- status post uterine fibroid resection. C-section x 1. s/p Tubal ligation.

Social history – Never smoker. Occasional EtOH < 1 drink per week. Married, has one child.

Family History – mother died of colon cancer at age 62. Father died of lung cancer at age 71. No family history of breast cancer.

Objective:

Vitals: 98.5, HR – 83 bmp, RR- 16 bpm, BP – 105/58 mmHg. 98% on room air.

Gen- well appearing, no acute distress

Neck – no cervical or supraclavicular lymphadenopathy

Breast – no palpable nodules. No overlying skin redness.

Lungs – no use of accessory muscles. clear to auscultation bilaterally.

CV- regular rate and rhythm with normal S1/S2 and no m/r/g

Abdominal – soft, non-distended, bowel sounds present, NTTP in all 4 quadrants. No hepatosplenomegaly

Extremities- no edema

Lymph- no axillary or cervical adenopathy palpated

Activity 2: Case discussion questions:

1. Briefly, discuss structured methods for generating a prompt.
2. Use the LLM to understand key components of the history, physical, and diagnostic tests to assess breast cancer risk.
3. Using best practices in prompt generation – ask the LLM to make a recommendation on the initial mode of breast cancer screening for this patient. If students have access, have them try different LLMs (Open-Evidence vs. Gemini or OpenAI, or others).
4. Apply a structured approach to appraise AI outputs using Sackett’s 5-step framework for evidence-based practice.
5. Ask the LLM what references it used to make its decision, and verify those references. Did anyone obtain hallucinated references?
6. Is the strength of the recommendation from the AI tool consistent with the guidelines? (guidelines provided at the table, Canvas, or PubMed link below)

| **EBP Step** | **Application to AI Use** | **Example Clinical Question** |
| --- | --- | --- |
| 1. Ask the question | Formulate a clear, answerable question using a structured prompt | In a 47-year-old female with heterogeneously dense breast tissue, and no family history of breast cancer, what is the optimal test for initial breast cancer screening? |
| 2. Acquire information | Use the AI tool to retrieve relevant evidence, guidelines or summaries. | Summarize and contrast USPSTF, Oncology, and Radiology guidelines comparing recommendations? |
| 3. Appraise | Critically evaluate the AI output for accuracy and alignment with the standard of care. Compare with trusted sources. | Does the AI-recommended strategy match UpToDate or guideline-based recommendations? |
| 4. Apply findings to practice | Integrate the AI-outputs with clinical expertise and patient values. | Would this strategy be appropriate for this patient given their greater fear of a false negative than a false positive? |
| 5. Assess outcomes | Reflect on the outcome and the AI tool’s utility. Consider revising prompts or finding alternative sources. | Did the AI output improve decision-making or require significant correction? Patient outcome? |

*Table: author owned*

**Case Continued**

Imaging – Digital Mammography and bilateral digital 3D tomosynthesis and a limited US was performed. And synthetic 2D digital mammography images were obtained. Computer-aided R2 CAD.

- 1.4 x 0.8 x 1.0 cm nodule in the left breast at 7 o'clock, which persists on focal compression. BI-RADS IV- suspicious finding. Heterogeneously dense breast tissue.
- On focused US – hypoechoic parallel mass with irregular margins. No significant internal vascularity. US of the left axilla shows normal lymph nodes.

Vignette prior to activity 3:

Based on the results of a competition to yield improved algorithms for identifying cancerous breast lesions on DBT, your hospital has adopted a machine learning algorithm that predicts malignant vs. benign lesions. The algorithm has a sensitivity of 0.96 for detecting cancerous lesions on biopsy.

**Optional reference and link**: *Konz N, Buda M, Gu H, et al. A Competition, Benchmark, Code, and Data for Using Artificial Intelligence to Detect Lesions in Digital Breast Tomosynthesis. JAMA Netw Open. 2023;6(2):e230524. Published 2023 Feb 1. doi:10.1001/jamanetworkopen.2023.0524*

[A Competition, Benchmark, Code, and Data for Using Artificial Intelligence to Detect Lesions in Digital Breast Tomosynthesis - PubMed](https://pubmed.ncbi.nlm.nih.gov/36821110/)

The AI algorithm predicts the patient’s lesion as benign, but the attending's clinical experience leads them to recommend a biopsy in accordance with standard guidelines for managing a BI-RADS IV nodule.

Activity 3: Case (cont.) discussion questions:

1. Use a generative AI application of your choosing. How should this patient’s DBT and US be followed up? What additional testing could be performed? What ethical concerns must be addressed when incorporating AI into this patient care decision?
2. When such a machine learning algorithm assists in the interpretation of biopsy results within the local healthcare system, what ethical issues arise? (consider bias, transparency, consent). What factors influence the decision to adopt such tools?
3. How do you inform the patient about any potential discordance between AI and human recommendations for the follow-up plan for the biopsy?
4. Can the LLM adjust recommendations based on patient preference? Try additional prompts that incorporate variations of patient preferences.

**Part 3: Synthesis case conclusion and debrief**

Part three contains the case conclusion and debriefing session for this synthesis session. What aspects of integrating AI into this case seemed useful? What ethical challenges arose from integrating AI outputs into this patient’s care?

The patient underwent a biopsy, which revealed a Fibroadenoma without signs of malignancy. The patient’s loss of her parents due to other cancer types and young children, as well as independent reading on the impact of dense breast tissue, strongly impacted her own perceptions of risk vs. benefits of additional management. The Surgical Oncologist and Breast Radiologist recommend an excisional biopsy due to the discrepancy between her nodule imaging characteristics and biopsy results.  After a discussion with the Breast Radiologist and Surgical Oncologist, she opts to proceed with an excisional biopsy.

Excisional biopsy results:
• Excisional biopsy- Fibroadenomatoid change. Background sclerosing adenosis and microcalcification. Negative for in situ or invasive carcinoma

Patient has a stable post-op course and is recommended to resume routine screening.

Activity 4: Debrief: These questions are suggestions and additional questions can be generated by the facilitator based on the group’s discussion and interest.

1. As first-year students, what was the most useful task the LLM performed? Guidance on the important risk factors and data to collect when seeing the patient? Or the potential to help make individual patient care decisions? Or simply time to read more about breast cancer screening and obtaining reference sources to guide learning?
2. What are your biggest concerns as you navigate the role of managing AI predictions or patient care recommendations? (Do you acknowledge this as being “high-stakes” AI use?). How can we as educators address those concerns?
3. As a student, you may consider using AI outputs both to learn and to assist in generating a care plan. These different roles carry different responsibilities and risks with respect to managing the AI output. Reflect on the slightly different roles. Have you found anything effective when using AI to enhance your own learning as opposed to risking “cognitive offloading” or atrophy of your critical thinking skills?
4. Given AI’s current environmental footprint and power requirements, should AI be used for clinical care recommendations when readily available access to guidelines or clinical risk calculators already exist?
5. Auditing AI output to ensure accuracy and “human-in-the-loop” decision-making takes time; is it worth the time? Or does the efficiency loss of this verification process guide you towards using other reference materials?
