# Supplementary material for: Introduction to Fundamental AI and Ethical Considerations: A Didactic and Interactive Case-Based Session for Preclerkship Medical Students
Source: MedEdPORTAL. 2026 Oct 23;22:11645. doi: 10.15766/mep_2374-8265.11645 (PMC13691575; doi:10.15766/mep_2374-8265.11645)
Supplement: Supplementary file 1 [file mep_2374-8265.11645-s001.zip › E. Postsession Survey.docx]

**Appendix E: Postsession survey, multiple-choice quiz, and distractor analysis**

Thank you for taking the time to complete this anonymous survey. Please answer each question honestly and to the best of your ability—your feedback is valuable in helping us improve our Fundamentals of AI and Ethics in Artificial Intelligence Healthcare Applications Synthesis sessions. There are quiz questions below; they are not graded and do not contribute to your module grade. Your responses are anonymous and will be used for future curriculum planning.

Block 1

Q1 When a clinician uses an AI-based tool to help formulate a treatment plan, who holds the primary professional responsibility for the patient's outcome?

- The company that developed the AI tool (1)
- The hospital or clinic that implemented the AI system (2)
- The clinician who makes the final patient care decision (3)
- Responsibility is shared equally between the clinician and the AI tool (4)

Q2 You are going over an AI-assisted treatment plan with a patient and the patient asks, "How did this AI tool produce this recommendation?” The clinician finds this question difficult and is unable to find information relating to the AI tool's development and validation. The clinician's difficulty answering this patient's question relates most closely with which of the following principal issues relating to ethical AI use?

- Informed consent (1)
- Safety in AI tool outputs (2)
- Transparency in shared decision making (3)
- Data privacy (4)

Q3 What is a primary factor that can introduce or perpetuate bias in AI-based healthcare algorithms?

- The processing speed of the computer hardware running the algorithm (1)
- The personal opinions and biases of the end-user clinicians interacting with the tool (2)
- Making AI "explainable" through transparency in the AI tools' internal and external validation processes (3)
- If certain populations are underrepresented in the datasets used to train and validate the tool (4)

Q4 When interacting with a generative AI tool to aid clinical decision making, which of the following is a best practice generating a prompt? Select the best response

- Define the task and intent of the prompt broadly, in order to not have the AI tool provide too narrow a response (1)
- Providing specific role, task, clinical context, audience, and the desired format for the answer (2)
- Focusing the Intent of the prompt on generating a single, definitive answer with minimal clinical context (3)
- Combining distinct Tasks and Audiences into a single prompt (e.g., asking for a clinical summary and a patient education handout simultaneously) (4)

Q5 When applying an AI tool to aid the development of a patient’s management plan, all of the following are essential considerations, except one?

- The current standard of care and patient’s preferences towards their own treatment (respect for autonomy in the decision) (1)
- The safety of the AI tool at other institutions during its initial validation (2)
- The clinician’s own judgement (3)
- If AI treatment recommendations falls within the clinician’s scope of practice (4)

Q6 *Before these sessions,* rate your knowledge in defining basic AI terminology?

- Not knowledgeable at all (1)
- Slightly knowledgeable (2)
- Moderately knowledgeable (3)
- Very knowledgeable (4)
- Extremely knowledgeable (5)

Q7 *After these sessions*, rate your knowledge in defining basic AI terminology?

- Not knowledgeable at all (1)
- Slightly knowledgeable (2)
- Moderately knowledgeable (3)
- Very knowledgeable (4)
- Extremely knowledgeable (5)

Q8 *Before these sessions*, rate your knowledge applying a structured approach towards generating a prompt to answer a medical question?

- Not knowledgeable at all (1)
- Slightly knowledgeable (2)
- Moderately knowledgeable (3)
- Very knowledgeable (4)
- Extremely knowledgeable (5)

Q9 *After these sessions*, rate your knowledge applying a structured approach towards generating a prompt to answer a medical question?

- Not knowledgeable at all (1)
- Slightly knowledgeable (2)
- Moderately knowledgeable (3)
- Very knowledgeable (4)
- Extremely knowledgeable (5)

Q10 Before these sessions, how knowledgeable were you in your ability to summarize factors that can bias or mitigate bias of AI outputs?

- Not knowledgeable at all (1)
- Slightly knowledgeable (2)
- Moderately knowledgeable (3)
- Very knowledgeable (4)
- Extremely knowledgeable (5)

Q11 After these sessions, how knowledgeable are you in your ability to summarize factors that can bias or mitigate bias of AI outputs?

- Not knowledgeable at all (1)
- Slightly knowledgeable (2)
- Moderately knowledgeable (3)
- Very knowledgeable (4)
- Extremely knowledgeable (5)

Q12 *Before these sessions*, how knowledgeable were you in your ability to summarize the ethical principles that apply to AI applications used in patient care settings?

- Not knowledgeable at all (1)
- Slightly knowledgeable (2)
- Moderately knowledgeable (3)
- Very knowledgeable (4)
- Extremely knowledgeable (5)

Q13 *After these sessions*, how knowledgeable are you in your ability to summarize the ethical principles that apply to AI applications used in patient care settings?

- Not knowledgeable at all (1)
- Slightly knowledgeable (2)
- Moderately knowledgeable (3)
- Very knowledgeable (4)
- Extremely knowledgeable (5)

Q14 *Before these sessions*, rate your knowledge applying a structured approach to assess accuracy and quality of AI-based outputs for medical questions?

- Not knowledgeable at all (1)
- Slightly knowledgeable (2)
- Moderately knowledgeable (3)
- Very knowledgeable (4)
- Extremely knowledgeable (5)

Q15 *After these sessions*, rate your knowledge applying a structured approach to assess accuracy and quality of AI-based outputs for medical questions?

- Not knowledgeable at all (1)
- Slightly knowledgeable (2)
- Moderately knowledgeable (3)
- Very knowledgeable (4)
- Extremely knowledgeable (5)

Q16 What did you like about the current sessions? (Open-ended)

________________________________________________________________

________________________________________________________________

Q17 What about these sessions could be improved for next year? (Open-ended)

________________________________________________________________

________________________________________________________________

**Appendix E: Distractor analysis for 5 question MCQ.**

The purpose of presenting this is for educators to use this data to make any edits or additions that would made potential quiz items more transferable to local institutional context.

| Item | Option | Prop_Upper | Prop_Lower | Distractor_Discrimination | PointBiserial |
| --- | --- | --- | --- | --- | --- |
| Q1 | C | 0.923 | 0.769 | -0.154 | 0.089 |
| Q1 | D | 0.077 | 0.115 | 0.038 | 0.104 |
| Q1 | B | 0 | 0.038 | 0.038 | 0.01 |
| Q1 | A | 0 | 0.077 | 0.077 | -0.443 |
| Q2 | B | 0 | 0.577 | 0.577 | -0.022 |
| Q2 | C | 1 | 0.346 | -0.654 | 0.027 |
| Q2 | A | 0 | 0.077 | 0.077 | -0.047 |
| Q2 | D | 0 | 0 | 0 | 0.096 |
| Q3 | B | 0 | 0.346 | 0.346 | -0.178 |
| Q3 | D | 1 | 0.385 | -0.615 | 0.322 |
| Q3 | C | 0 | 0.154 | 0.154 | 0.006 |
| Q3 | A | 0 | 0.115 | 0.115 | -0.422 |
| Q4 | B | 1 | 0.692 | -0.308 | 0.365 |
| Q4 | A | 0 | 0.231 | 0.231 | -0.431 |
| Q4 | D | 0 | 0.077 | 0.077 | 0.023 |
| Q5 | B | 0.923 | 0.231 | -0.692 | 0.005 |
| Q5 | D | 0.038 | 0.654 | 0.615 | 0.059 |
| Q5 | A | 0.038 | 0.077 | 0.038 | -0.208 |
| Q5 | C | 0 | 0.038 | 0.038 | 0.089 |

| When a clinician uses an AI-based tool to help formulate a treatment plan, who holds the primary professional responsibility for the patient's outcome? | | | | |
| --- | --- | --- | --- | --- |
| Item 1 |  | # | Distractor performance | |
| A | company | 2 | poor |  |
| B | hospital | 1 | poor |  |
| C | **clinician** | 85 |  |  |
| D | responsibility | 10 | high performing distractor | |
|  |  |  |  |  |
|  |  |  |  |  |
| You are going over an AI-assisted treatment plan with a patient and the patient asks, "How did this AI tool produce this recommendation? The clinician finds this question difficult and is unable to find information relating to the AI tool's development and validation. The clinician's difficulty answering this patient's question relates most closely with which of the following principal issues relating to ethical AI use? | | | | |
| Item 2 |  | # |  |  |
| A | Informed consent | 8 | high performing distractor | |
| B | safety in AI | 27 | high performing distractor | |
| C | **transparency** | 62 |  |  |
| D | data privacy | 1 | unhelpful distractor | |
| What is a primary factor that can introduce or perpetuate bias in AI-based healthcare algorithms? | | | | |
| Item 3 |  |  |  |  |
| A | processing speed… | 3 | good |  |
| B | personal opinions… | 12 | good |  |
| C | making AI explainable… | 5 | not a good distractor | |
| D | **underrepresented populations…** | 78 |  |  |
| When interacting with a generative AI tool to aid clinical decision making, which of the following is a best practice generating a prompt? Select the best response | | | | |
| Item 4 |  | # |  |  |
| A | Define broadly… | 6 | good |  |
| B | **Provide specific…** | 90 |  |  |
| C | focusing the intent… | 0 | Unhelpful distractor |  |
| D | Combine… | 2 | Unhelpful distractor |  |
|  |  |  |  |  |
| When applying an AI tool to aid the development of a patient’s management plan, all of the following are essential considerations, except one? | | | | |
| Item 5 |  | # |  |  |
| A | current standard… | 5 | helpful |  |
| B | **safety of AI tool…** | 55 |  |  |
| C | Clinicians judgement… | 3 |  |  |
| D | scope of practice… | 35 | Unhelpful distractor | |
